# 2022: A landmark year for *JOR Spine*

**DOI:** 10.1002/jsp2.1239

**Published:** 2022-12-29

**Authors:** Mauro Alini, Robert Mauck, Daisuke Sakai

**Affiliations:** ^1^ AO Research Institute, AO Foundation Davos Switzerland; ^2^ McKay Orthopaedic Research Laboratory University of Pennsylvania Philadelphia Pennsylvania USA; ^3^ Department of Orthopaedic Surgery Tokai University School of Medicine Tokyo Japan

Dear Readers,

As the 2022 year is coming to its end, we would like again to thank all who contributed to JOR Spine achieving a first excellent impact factor of 3.75. This ranks our Journal second within the spine category, and as the go‐to place to submit your best spine research. This achievement has already increased the submission rate to the journal, with a record number of submitted manuscripts in 2022.

Another important scientific event just concluded: the 6th International Philadelphia Spine Research Symposium (PSRS) at the Skytop Lodge, in Pennsylvania. This was an amazing and very successful get‐together after 3 years of spine research starvation. With a great participation (more than 200 attendees), outstanding keynote lectures, remarkable general presentations (especially from well‐prepared young investigators), profitable networking sessions, a beautiful location, and great afternoon events, with a wonderful weather, for sure it was the best conference of the last several years. We would like to thank the organizers, Prof *Makarand V. Risbud*, Prof *Lachlan Smith*, and Prof *Irving Shapiro*, as well as the whole PSRS team, including the ORS staff for the excellent organization. We were so happy to join together as a community once again and are very much looking forward for the 2024 venue! Also, we are again planning a *JOR Spine*‐PSRS special issue for the coming year, highlighting the best scientific presentations from the meeting.

Last but not least, we are pleased to present to you the *JOR Spine* Early Career Award 2022 winners. This Award is designed to recognize the “rising stars” of our field and highlight their outstanding work published in the *JOR Spine*. This year's competition included a number of really outstanding candidates, making selection of the winner very difficult. We are delighted to acknowledge and congratulate Dr *Rebecca Wachs* and Dr *Benjamin (Ben) Walter* as the winners of this year's ORS Spine Section/JOR Spine Early Career Award. We look forward to another exciting competition for next year—please keep your eyes out for the announcement and spread the word to emerging leaders in the spine community.

Finally, we hope to see all of you at the next ORS meeting in Dallas, especially at the ORS Spine Section event, scheduled for Friday afternoon on February 10, 2023.

Thank you all again for your incredible support this past year. In 2023, let us continue our work together to bring *JOR Spine* where it should be, at the top of the spine journal pyramid.

With our best wishes for wonderful seasonal festivities.

Mauro, Rob, and Dai.

